# Embryotoxicity Induced by Triclopyr in Zebrafish (*Danio rerio*) Early Life Stage

**DOI:** 10.3390/toxics12040255

**Published:** 2024-03-29

**Authors:** Ítalo Bertoni, Bianca Camargo Penteado Sales, Cristina Viriato, Paloma Vitória Lima Peixoto, Lílian Cristina Pereira

**Affiliations:** 1 Medical School, São Paulo State University (Unesp), Botucatu 18618-687, Brazil; bianca.cp.sales@hotmail.com (B.C.P.S.); paloma.peixoto@unesp.br (P.V.L.P.); 2 Center for Evaluation of Environmental Impact on Human Health (TOXICAM), Botucatu 18618-687, Brazil; cristina.viriato@unesp.br (C.V.); lilian.pereira@unesp.br (L.C.P.); 3 Institute of Biosciences, São Paulo State University (Unesp), Botucatu 18618-689, Brazil; 4 School of Agriculture, São Paulo State University (Unesp), Botucatu 18610-034, Brazil

**Keywords:** triclopyr, contaminant of emerging concern, zebrafish, new methodology approach, embryotoxicity

## Abstract

Triclopyr, an auxin-like herbicide that is widely employed for managing weeds in food crops and pastures, has been identified in various environmental settings, particularly aquatic ecosystems. Limited understanding of the environmental fate of this herbicide, its potential repercussions for both the environment and human health, and its insufficient monitoring in diverse environmental compartments has caused it to be recognized as an emerging contaminant of concern. In this study, we have investigated how triclopyr affects zebrafish, considering a new alternative methodology. We focused on the endpoints of developmental toxicity, neurotoxicity, and behavior of zebrafish embryos and larvae. We determined that triclopyr has a 96 h median lethal concentration of 87.46 mg/L (341.01 µM). When we exposed zebrafish embryos to sublethal triclopyr concentrations (0.5, 1, 5, 10, and 50 μM) for up to 144 h, we found that 50 µM triclopyr delayed zebrafish egg hatchability. Yolk sac malabsorption was significant at 0.5, 1, 5, and 10 µM triclopyr. In zebrafish larvae, uninflated swim bladder was significant only at 50 µM triclopyr. Furthermore, zebrafish larvae had altered swimming activity after exposure to 10 µM triclopyr for 144 h. In summary, these comprehensive results indicate that even low triclopyr concentrations can elicit adverse effects during early zebrafish development.

## 1. Introduction

Triclopyr (2-(3,5,6-trichloropyridin-2-yl)oxyacetic acid) is a highly effective auxin-like herbicide. It is extensively used as a pre-emergence herbicide in crops such as corn, soybean, and wheat, and it is applied as a post-emergence herbicide in pastures and rice, eucalyptus, and soy crops to manage broadleaf weeds and woody plants [1,2]. Moreover, it is employed in urban environments to control weeds and plants [1]. Triclopyr is available in two commercial formulations, namely butoxyethyl ester (Triclopyr BEE) and triethylamine salt (Triclopyr TEA) [1]. It displays low mammalian toxicity via the acute oral route, with a 96 h median lethal dose (LD50) of 630–729 mg/kg in rats [3]. In Brazil, the Maximum Residue Limit (MRL) of triclopyr in agricultural products is 0.07 mg/kg [2]. Additionally, a Dietary Ingestion Acceptable (DIA) value of 0.03 mg/kg has been set by the European Food Safety Authority [4] to ensure that food products containing triclopyr residues are safe for consumption.

Triclopyr exhibits high water solubility (440 mg/L), so it can penetrate aquatic environments after it is applied. Consequently, this herbicide is frequently detected in various aquatic matrixes, which underscores the extent of water contamination attributed to it [1]. Groundwater analysis has revealed triclopyr levels ranging from 0.006 to 0.58 parts per billion (ppb) [5]. Rawn et al. [6] reported triclopyr concentrations ranging from 0.50 to 33.4 nanograms per liter (ng/L) in rivers. Particularly, water bodies near agricultural fields have been shown to present elevated triclopyr levels surpassing 2 milligrams per liter (mg/L) [7]. Estuaries have been demonstrated to contain a maximum triclopyr concentration of 0.079 micrograms per liter (μg/L) [8], and wastewater effluents have been shown to contain 620 ng/L triclopyr [9].

Triclopyr is stable to abiotic hydrolysis, and its degradation at pH 5, 7, and 9 is negligible over 30 days [10]. Besides that, triclopyr is resistant to photolysis in soil and remains essentially unchanged by anaerobic aquatic metabolism, boasting a half-life exceeding 1000 days [11]. These comprehensive findings contribute to our understanding of the presence and persistence of triclopyr in diverse aquatic environments [12].

Previous toxicity studies have indicated that triclopyr has a 96 h median lethal concentration (LC50-96h) of 117 mg/L in Rainbow trout (*Oncorhynchus mykiss*) [3]. Triclopyr LC50-96h ranging from 5.3 to 9.7 mg/L has also been reported for different species of juvenile salmonids [13]. Kreutzweiser et al. [14] illustrated acute lethal effects in fish following exposure to triclopyr. In addition, sublethal effects, including disorientation, coughing, and gill surface widening, have been observed in Rainbow trout (*Oncorhynchus mykiss*) and King salmon (*Oncorhynchus tshawytscha*) exposed to triclopyr [14]. A study conducted by Guilherme et al. [15] investigated the triclopyr genotoxic effects in European eel (*Anguilla anguilla* L.) fish using the comet assay, which revealed triclopyr-induced genotoxicity in this fish species when it was exposed to 30 or 120 µg/L triclopyr over a short time [15]. However, the actual mechanisms through which triclopyr exerts toxicity to non-target aquatic organisms are unclear. Limited knowledge of triclopyr toxicity during early fish development, mainly embryotoxicity and neurotoxicity induced by low triclopyr concentrations, highlights a critical gap. Early life impacts, especially during embryonic development, can shape the future performance of organisms at the molecular and population levels. Early exposure to contaminants may have extensive consequences, impacting the overall health, molecular processes, and population dynamics of fish species beyond immediate effects [16].

Fish serve as suitable organisms for research because they play vital roles in the aquatic food chain and are efficacious bioindicators. Their heightened sensitivity to aquatic pollutants further underscores their importance in ecotoxicological studies [16]. Zebrafish (*Danio rerio*), freshwater teleost cyprinid fish, have emerged as a pivotal predictive and high-throughput model in toxicological research [17]. Zebrafish are appealing as an organism model because they are small, easy to handle and breed in large numbers, and transparent during early development [18]. Moreover, zebrafish and humans have similar xenobiotic metabolism [19], which allows researchers to extrapolate and to gain deeper insight into how potentially toxic substances may impact human health. In addition, zebrafish are highly sensitive to herbicide toxicity [20], providing valuable insight into how these compounds affect aquatic ecosystems and human health, thereby fostering a safer and more sustainable environment [21].

While other studies have previously given consistent evidence of triclopyr toxicity to other aquatic species, here we aimed to enhance our understanding of its toxicity. We have employed zebrafish in early life stages as a new methodology approach (NAM) and used different endpoints to establish the toxicity mechanism of triclopyr, including its developmental toxicity, its neurotoxicity assessed through acetylcholinesterase (AChE) activity analysis, and its ability to modify zebrafish behavior. The toxicity tests were conducted within a range of and above triclopyr concentrations that have previously been identified and documented in the environment [6,7,8,9]. This approach aimed to broaden the range of responses and to identify effect thresholds, offering novel insights into the mode of action of triclopyr and its impact on non-target aquatic organisms.

## 2. Materials and Methods

### 2.1. Chemicals and Test Solutions

Triclopyr (CAS: 55335-06-3, purity 99.9%; Product No 32016) was purchased from Sigma-Aldrich. The stock triclopyr solutions were prepared with reconstituted water (2 mM CaCl_2_, 0.5 mM MgSO_4_, 0.75 mM NaHCO_3_, and 0.07 mM KCl in ultrapure water) according to the ISO 7346-1 guideline [22]. 3,4-Dichloroaniline (3,4-DCA) (CAS: 95-76-1, purity 98%; Product No 437778), Bradford’s reagent (Product No B6916), 5,5-dithio-bis-(2-nitrobenzoic acid) (CAS: 69-78-3; Product No D8130), acetylthiocholine iodide (CAS: 2260-50-6, purity ≥97%; Product No A7000), and dichlorvos (CAS: 62-73-7, purity 98.9%; Product No 45441) were purchased from Sigma-Aldrich.

### 2.2. Zebrafish Husbandry and Egg Collection

Adult wild-type zebrafish (AB strain) were sourced from the vivarium at the Medical School of Botucatu and raised under controlled conditions in a recirculating aquaculture system. The system maintained a constant temperature of 27 ± 1 °C, pH at 7.0 ± 0.5, dissolved oxygen levels at 6 mg/L, conductivity of 750 ± 50 μS/cm, and a light/dark cycle of 14/10 h. Zebrafish were provided with three daily feedings consisting of dry food (Maramar^®^ Tropical Granules, Lima, Perú; Alcon^®^ Spirulina Ration, Geneva, Switzerland) and live food (*Artemia salina*, Maramar^®^).

In preparation for egg collection, during the late afternoon of the day before the test was initiated, groups of male and female zebrafish in a 2:1 ratio were individually placed within the spawning tank and maintained under these conditions until the following morning. Eggs were collected immediately after spawning and rinsed with distilled water, to remove impurities. Subsequently, the eggs were transferred to Petri dishes containing reconstituted water [22]. Fertilized and healthy eggs of zebrafish at ≤3 h post-fertilization (hpf) were selected with the assistance of an inverted microscope (AE2000, Motic^®^, Barcelona, Spain). Unfertilized, coagulated, or non-viable eggs were discarded following good laboratory practice (GLP) and the guidelines of the National Council for the Control of Animal Experimentation (CONCEA). All the experiments were conducted with the approval of the Ethics Committees on Animal Use of the Botucatu School of Medicine (CEUA-UNESP) and the University of São Paulo (CEUA-USP), under certificates numbered 1300/2019 and 22.1.270.60.5, respectively.

### 2.3. Chemical Exposure and Development Toxicity Assessment

The zebrafish embryo acute toxicity test was conducted in accordance with the Organization for Economic Co-operation and Development (OECD) Guideline for Testing Chemicals, Test No. 236: Fish Embryo Acute Toxicity (FET) Test [23], with slight modifications. To determine the triclopyr LC50, 30 zebrafish embryos ≤ 1 hpf were distributed into 24-well plates; 5 embryos were placed in each well containing 2 mL of triclopyr at a certain concentration, namely 194.95, 231.84, 275.70, 327.87, or 389.90 µM. Reconstituted water was used as control, and 4 mg/L 3,4-DCA was employed as lethality control to assess mortality rates [22,23]. To ensure triclopyr stability, the test medium was renewed every 48 h until the experiment was completed. The plates were kept in an incubator (SSB, SolidSteel, Città Sant’Angelo, Italy) at a constant temperature of 27 ± 1 °C under a light/dark cycle of 14/10 h. The lethality criteria were defined in accordance with OECD TG236 [23], which assesses embryo coagulation, lack of somite formation, non-detached tail, and absence of heartbeat. These parameters were examined every 24 h using an inverted microscope. The GraphPad Prism software version 8.0.2 was employed to calculate the triclopyr LC50-96h.

To assess the effects of triclopyr at environmentally relevant concentrations (below LC50), 20 healthy zebrafish embryos (≤1 hpf) were exposed to each sublethal concentration, 0.5, 1, 5, 10, or 50 µM triclopyr, in 24-well plates (one embryo per well). Reconstituted water and 4 mg/L 3,4-DCA were employed as controls [22,23]. The plates were kept in an incubator at 27 ± 1 °C under a light/dark cycle of 14/10 h. The test medium was renewed every 48 h until the experiment was completed. Each exposure experiment was performed in independent triplicates. The embryos were evaluated under an inverted microscope at 24, 48, 72, 96, 120, and 144 hpf. Hatchability was analyzed by calculating the ratio of hatched embryos to the total number of embryos for each treatment until 144 hpf. The ratio of swim bladder development was determined by calculating the proportion of larvae with inflated swim bladders to the total number of embryos exposed to triclopyr up to 144 hpf. The swim bladder analyses were conducted at 96 hpf, 120 hpf, and 144 hpf under an inverted microscope.

### 2.4. Morphological Assessment

For morphological assessment, twenty healthy embryos were exposed to 0.5, 1, 5, 10, or 50 µM triclopyr (20 embryos per plate for each replicate of an experimental group, with one embryo per well); OECD Test No. 236: Fish Embryo Acute Toxicity (FET) Test was followed [23]. At 96 hpf, 15 larvae from each treatment group were individually transferred onto concave glass slides and anesthetized with 4 µg/mL tricaine (MS-222, Sigma Aldrich, St. Louis, MO, USA). Images of the larvae at 96 hpf were captured with a digital camera (AxioCam ICc 5, Zeiss, Jena, Germany) attached to a stereomicroscope (Stemi 508, Carl Zeiss, Oberkochen, Germany). Morphological measurements were conducted using the DanioScope software (version 1.1, Noldus Information Technology, Wageningen, The Netherlands). The test was performed in triplicate with independent trials. Four morphometric parameters were evaluated for each zebrafish larva, including body length (µm), eye size area (µm^2^), pericardial area (µm^2^), and yolk sac area (µm^2^). To compare the treated organisms, the description of normal zebrafish development provided by Kimmel [24] was used as reference.

### 2.5. Locomotor Activity

The locomotor activity assay was performed according to a previous study of Abe et al. [25]. To assess locomotor activity of zebrafish in the early life stage following exposure to triclopyr, three sublethal triclopyr concentrations (0.5, 5, or 10 µM), close to environmental detection levels [6,7,8,9], were selected, along with a control (reconstituted water). For each treatment condition, 24 viable eggs (≤3 hpf) were selected and randomly placed in six-well plates (8 eggs per well); each well was filled with 10 mL of the respective test solution. The plates were incubated at a constant temperature of 27 ± 0.5 °C under a light/dark cycle of 14/10 h. At 144 hpf, the zebrafish larvae were analyzed under a stereomicroscope. Only larvae exhibiting visually normal morphology and active swimming behavior, which indicate swim bladder development, were chosen for the experiment. Before locomotion was tracked, the zebrafish larvae were transferred to a 96-well plate (*n* = 24 larvae per treatment, 1 larva per well filled with 300 μL of reconstituted water) and allowed to acclimate at a temperature of 27 ± 0.5 °C for 60 min. The larval locomotor activity was analyzed using the ZebraBox and ZebraLab tracking system (Viewpoint Life Sciences, Lyon, France), in independent triplicates. Free swimming activities were monitored during a cycle consisting of four alternating periods: 10 min of light (stimulus with LED illumination) followed by 10 min of darkness (infrared illumination), preceded by a 10 min acclimatization comprising 5 min of light cycle and 5 min of dark cycle. The total swimming distance (mm) and time the larvae remained in each period (s) were recorded. The following swimming parameters were considered: large and small distance moved (mm), total distance moved (mm), and time spent in movement (s). In addition, the duration of inactivity (s) was analyzed.

### 2.6. Acetylcholinesterase Activity

AChE plays a crucial role in regulating neurotransmission. AChE inhibition has been linked to neurotoxicity and disrupted biological organization at higher levels, including impaired behavior and development. Additionally, AChE is commonly employed as a sensitive biomarker for exposure to pesticides and pollutants, offering valuable insight into the biological response of aquatic organisms to chemical agents [26].

AChE activity was assessed by following the method described by Ellman et al. [27], adapted for a 96-well plate, in independent triplicates. First, 30 healthy fertilized zebrafish eggs (≤3 hpf) were randomly selected for each treatment condition. The eggs were exposed to triclopyr treatment solutions (0.5, 1, 5, 10, or 50 µM) and controls in a six-well plate (*n* = 30 eggs per treatment, 10 eggs per well). Each well contained 10 mL of the respective treatment solution (at a ratio of one egg per mL). The plates were incubated at a constant temperature of 27 ± 0.5 °C under a light/dark cycle of 14/10 h. Following exposure, a pool of 30 zebrafish larvae at 96 hpf from each treatment group was transferred to 1.5 mL microtubes and homogenized with 500 µL of ice-cold phosphate buffer (pH 7.2). The samples were centrifuged at 2500× *g* and 4 °C for 10 min, to obtain the supernatant. The supernatant was used to quantify proteins by the Bradford method [28], adapted for a 96-well plate, and measured on a microplate reader (Synergy HTX, BioTek, Winooski, VT, USA).

To determine the kinetics of AChE activity in the enzymatic assay, 50 µL of the supernatant sample was added to each well in triplicate, followed by the addition of 250 µL of the reaction buffer (0.075 M acetylcholine iodide, 17 mM sodium bicarbonate, and 5,5′-4.7 mM dithiobis(2-nitrobenzoic acid)). The reaction was monitored by measuring the absorbance of the samples at 414 nm every 20 s for 5 min. Dichlorvos at 50 µM was used as positive control. Enzymatic activity was expressed in nmol of acetylthiocholine hydrolyzed per minute per milligram of protein. For the calculations, a molar extinction coefficient of 13.6 × 10^3^ M^−1^ cm^−1^ was applied.

### 2.7. Statistical Analysis

All the data are presented as the standard error of the mean (SEM). Normal distribution of the data obtained from the tests was assessed using the Shapiro–Wilk test. To determine whether significant differences existed among the treatments, a one-way Analysis of Variance (ANOVA) was conducted, followed by Dunnett’s post hoc test. The behavioral analyses were conducted utilizing two-way ANOVA followed by Tukey’s post hoc test. Results with *p* ≤ 0.05 were considered significant. Analyses were performed using GraphPad Prism software version 8.0.2 (GraphPad Software, San Diego, CA, USA).

## 3. Results

### 3.1. FET Test (LC50) and Development Toxicity

**Figure 1:**
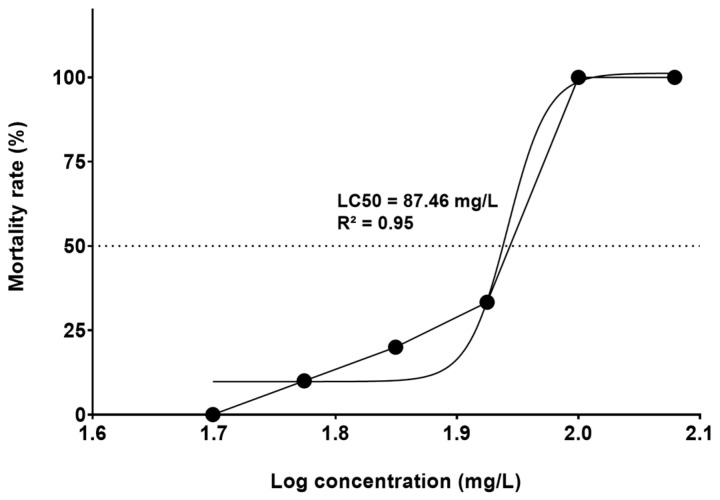
Determination of triclopyr 96 h median lethal concentration (LC50-96h) in zebrafish embryos. The graph illustrates the mortality rate of zebrafish embryos after exposure to various triclopyr concentrations. LC50-96h was calculated using the sigmoid curve fitting method, with triclopyr concentrations expressed in logarithmic scale.

The concentration of triclopyr LC50-96h was 87.46 mg/L (341.01 µM) (95% confidence interval ranging from 69.48 to 110.1 mg/L) (Figure 1). Throughout the test, there was no mortality in the control group. The group treated with 4 mg/L 3,4-DCA exhibited embryo mortality greater than 80% within the first 96 h, confirming that our testing procedure conducted according to OECD TG236 [23] was robust.

In the developmental toxicity test until 144 hpf, the control group displayed no mortality and had typical development, which agreed with the findings outlined by Kimmel et al. [24]. Throughout the test, there was no significant mortality in the groups treated with sublethal triclopyr concentrations.

**Figure 2:**
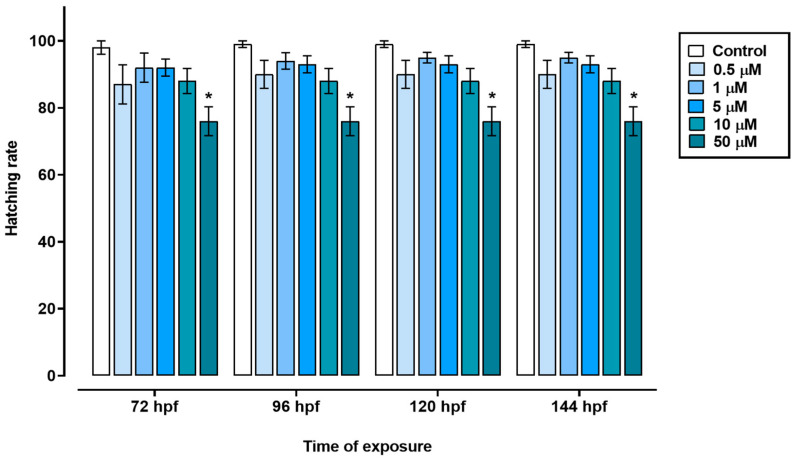
Hatching rate in zebrafish exposed to triclopyr. Effect of exposure to triclopyr on the hatching rate of zebrafish eggs in relation to exposure time. The proportion of hatched eggs by time and concentration is represented by different blue scales. The bars represent means, and the error bars represent the standard error of the mean (SEM), (*n* = 60). * One-way ANOVA followed by Dunnett’s test; *p* ≤ 0.05 for significant differences between exposed and control groups.

**Figure 3:**
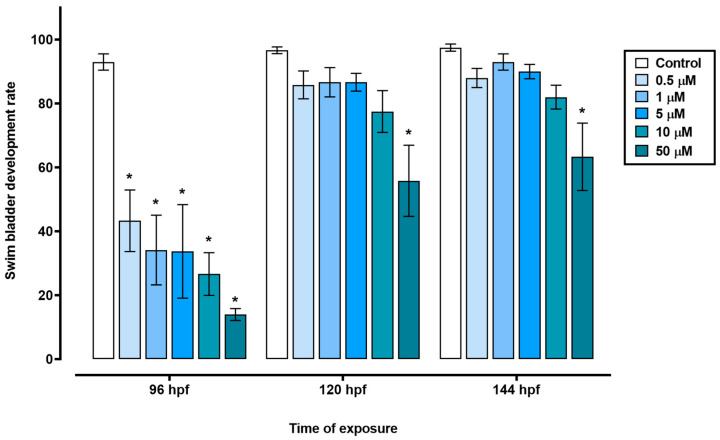
Swim bladder development in zebrafish exposed to triclopyr. Graphic representation of swim bladder development rate in zebrafish larvae by time of exposure to triclopyr. The proportion of swim bladder development by time and concentration is represented by different blue scales. The bars represent means, and the error bars represent the standard error of the mean (SEM), (*n* = 60). * One-way ANOVA followed by Dunnett’s test; *p* ≤ 0.05 for significant differences between exposed and control groups.

In the time frame of 72 to 144 hpf, the groups treated with 0.5, 1, 5, or 10 µM triclopyr and the control group showed similar egg hatching rates. However, the group exposed to 50 µM triclopyr had decreased egg hatching at 72 hpf, and this reduction persisted until 144 hpf (Figure 2). Furthermore, exposure to 0.5, 1, 5, 10, or 50 µM triclopyr (*p* ≤ 0.05) delayed swim bladder development, recorded at 96 hpf. Nevertheless, by the time the larvae reached 120 or 144 hpf, this delay was only significantly (*p* ≤ 0.05) evident in the group exposed to 50 µM triclopyr (Figure 3).

### 3.2. Morphological Analyses

**Figure 4:**
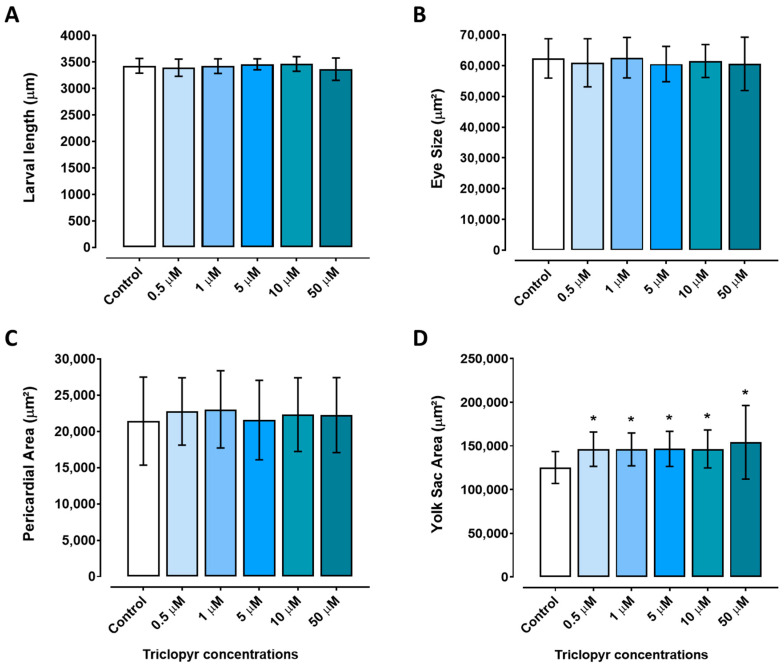
Morphological alterations induced by triclopyr in zebrafish at 96 h post-fertilization (hpf). (**A**) Larvae length; (**B**) eye size area; (**C**) pericardial area; (**D**) yolk sac area. The bars represent means, and the error bars represent the standard error of the mean (SEM), (*n* = 45). * One-way ANOVA followed by Dunnett’s test; *p* ≤ 0.05 for significant differences between exposed and control groups.

**Figure 5:**
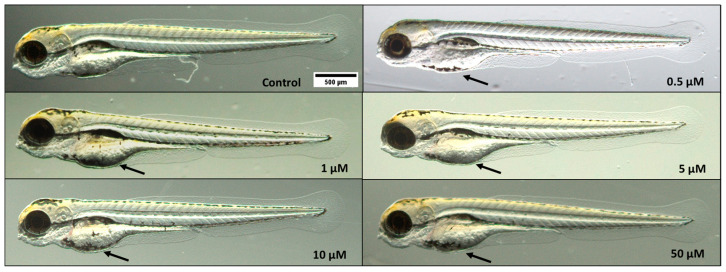
Effects of triclopyr on zebrafish larvae development at 96 h post-fertilization (hpf). Representative images of zebrafish larvae exposed to 0.5, 1, 5, 10, or 50 µM triclopyr, demonstrating noticeable yolk sac malabsorption (black arrow) in all treatment groups (magnification: 1.25×).

Embryos exposed to triclopyr at the tested concentrations for up to 96 h did not have a significantly reduced length compared to the control group (*p* ≤ 0.05) (Figure 4A). The eye area of zebrafish larvae was not altered when compared to the control group (Figure 4B). Similarly, the pericardium area was not different compared to the control group, but there was a suggestive trend toward an increase (Figure 4C). However, exposure to triclopyr resulted in retained yolk sac absorption (malabsorption) in zebrafish embryos, and there was a significant difference in the yolk sac area among all the triclopyr-treated groups and the control group (Figure 4D). Figure 5 illustrates the morphological comparison of zebrafish larvae at 96 hpf in the control and triclopyr-treated groups.

### 3.3. Locomotor Activity

**Figure 6:**
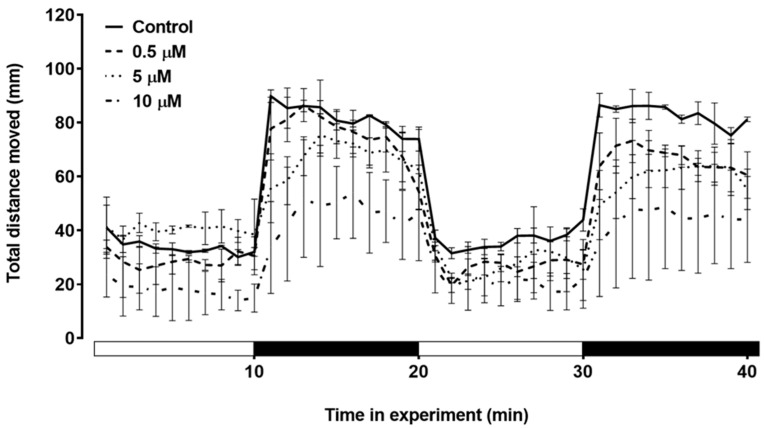
Total distance moved until 144 h post-fertilization (hpf) by zebrafish larvae exposed to triclopyr. Kinetics of the total distance (mm) moved by larvae during the light/dark stimulus. The lines represent different exposed groups, and the error bars denote the standard error of the mean (SEM).

**Figure 7:**
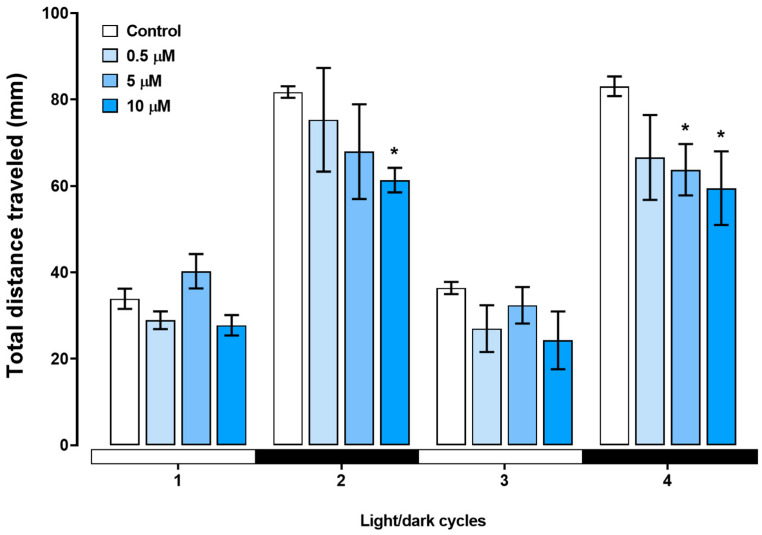
Total distance traveled until 144 h post-fertilization (hpf) in response to the light/dark stimulus test applied for larvae treated with triclopyr. The X-axis shows the whole experiment time and light/dark transition every 10 min. The bars represent means, and the error bars represent the standard error of the mean (SEM). * Two-way ANOVA multiple comparisons followed by Dunnett’s test, *p* ≤ 0.05 for significant differences between exposed and control groups.

We assessed the swimming behavior of zebrafish larvae during the light/dark transition. During the behavioral test, the zebrafish larvae exhibited a baseline pattern during the light cycles, and with the sudden transition to darkness, the larvae immediately showed an increase in locomotor activity. The kinetics of the total distance moved during the behavioral assay are depicted in Figure 6. In the first dark cycle, the larvae treated with 10 µM triclopyr showed a decrease in the total distance traveled compared with the control group (*p* ≤ 0.05). During the second dark stimulus, both the 5 µM and 10 µM triclopyr-treated groups demonstrated a significant decrease in the total distance traveled (*p* ≤ 0.05) compared to the control (Figure 7). No behavioral alterations were observed during the light cycles.

**Figure 8:**
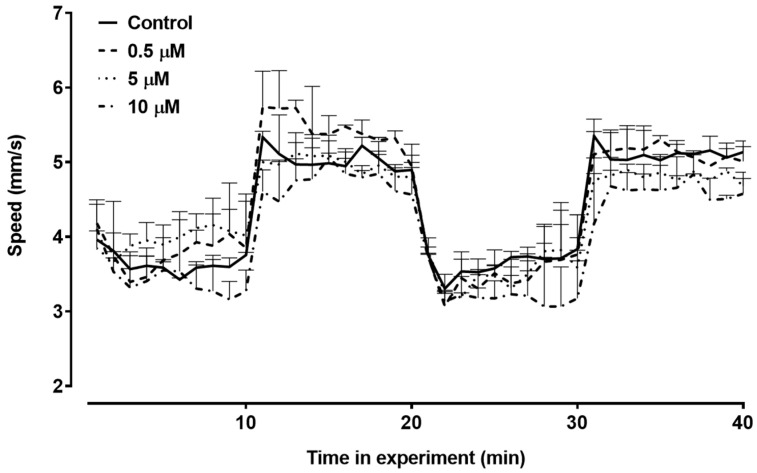
Swimming speed until 144 h post-fertilization (hpf) in zebrafish larvae exposed to triclopyr. The graph illustrates the kinetics of swimming speed (mm/s) exhibited by larvae during the light/dark stimulus. The lines represent different exposed groups, and the error bars denote the standard error of the mean (SEM).

We investigated how exposure to triclopyr impacted the average swimming speed of zebrafish larvae. The treated groups did not differ in terms of the mean swimming speed throughout this period (Figure 8). Therefore, under the conditions of this experiment, triclopyr did not induce changes in the average swimming speed of zebrafish larvae until 144 hpf.

### 3.4. Acetylcholinesterase Activity

**Figure 9:**
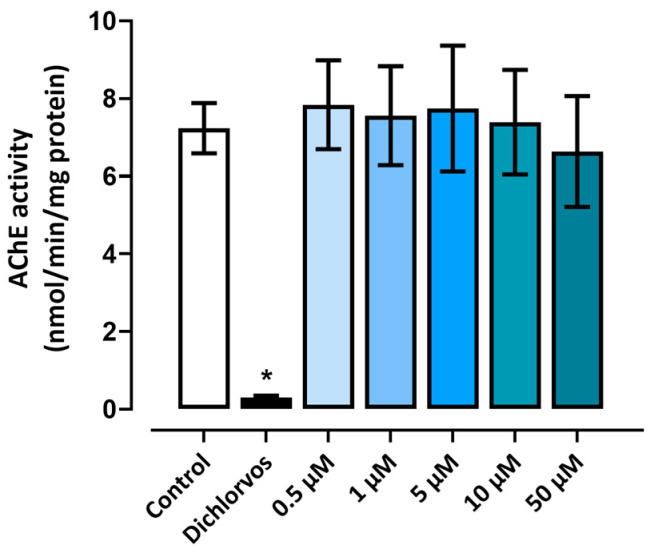
Acetylcholinesterase activity at 96 h post-fertilization (hpf) in zebrafish larvae exposed to triclopyr. AChE activity was determined in nmol per minute per milligram of protein. The bars represent means, and the error bars represent the standard error of the mean (SEM), (*n* = 3). * One-way ANOVA followed by Dunnett’s test; *p* ≤ 0.05 for significant differences between exposed and control groups.

We evaluated AChE activity at 96 hpf in zebrafish larvae. None of the groups treated with triclopyr showed significant results compared to the negative control. Thus, at the tested concentrations, triclopyr did not interfere in the AChE enzymatic functions in embryos or larvae. These results are shown in Figure 9.

## 4. Discussion

While some studies have demonstrated that triclopyr is safe in mammals from a reproductive and developmental standpoint [29], here we observed that exposing zebrafish embryos to triclopyr results in significant sublethal effects. These findings highlight the embryotoxic potential of triclopyr in zebrafish and its negative impacts on hatching rate, yolk sac absorption, swim bladder development, and swimming behavior. These discoveries underscore the importance of carefully evaluating the environmental effects of chemicals like triclopyr, particularly in sensitive aquatic organisms. Acute triclopyr toxicity to fish has previously been assessed, and outcomes such as mortality [13], as well as disorientation, coughing, and gill surface widening in adult fish have been evaluated [14]. Conversely, in a 96 h semi-static experimentation, Stehr et al. [30] did not note adverse effects in zebrafish embryos exposed to triclopyr concentrations of up to 10 mg/L (39 µM). However, in our study, we verified that triclopyr induces embryotoxicity during early zebrafish development, which is unprecedented among fish species exposed to triclopyr. During this critical period, the interplay of intrinsic and environmental factors, including environmental contaminants, can modify typical physiological and morphological traits [31].

According to Belanger et al. [32], LC50 values determined in assays conducted with zebrafish embryos (FET test) closely correspond to the values observed in traditional acute toxicity tests. In our study, we established LC50-96h of 87.46 mg/L (341.01 µM) for triclopyr in zebrafish embryos. Notably, for five juvenile Pacific salmonid species, including Coho salmon, Chinook salmon, Chum salmon, Pink salmon, Sockeye salmon, and Rainbow trout, triclopyr LC50-96h ranged between 5.3 and 9.7 mg/L (20.66 and 37.82 µM) [13]. Nevertheless, when we exposed zebrafish embryos to sublethal triclopyr concentrations within the same range, up to 50 µM (12.82 mg/L), we did not observe significant mortality. The resilience of zebrafish embryos to triclopyr concentrations that affect other fish species highlights the species-specific variations in sensitivity to this herbicide. These findings show that it is essential to conduct species-specific assessments to gain understanding of how triclopyr impacts aquatic organisms. This variability in sensitivity emphasizes the need for nuanced and tailored approaches in regulatory frameworks to safeguard different aquatic species from the potential adverse effects of exposure to triclopyr.

Egg hatching is crucial for zebrafish development and survival [24]. We verified delayed egg hatching in zebrafish embryos exposed to 50 µM triclopyr, with a 24% delay in the hatching rate between 72 and 144 hpf. Our results suggest that triclopyr induces structural or functional disturbances during zebrafish organogenesis, which may have modified the crucial physicochemical mechanisms for egg hatching in zebrafish embryos [33]. This observation aligns with the findings of Ahmad et al. [34], who reported a similar delay in the zebrafish egg hatching rate following exposure to 39 µM triclopyr for 96 h [34]. Additionally, similar to our findings, the auxin-like herbicides 2,4-D (2,4-dichlorophenoxyacetic acid) and dicamba also caused a reduction in zebrafish hatchability [35,36], which suggests that this outcome appears to be a notable characteristic of the toxicity induced by this class of herbicides in zebrafish. In contrast, Berrill et al. [37] did not observe any effect on egg hatching in bull frog (*Rana catesbeiana*), leopard frog (*Rana pipiens*), or green frog (*Rana clamitans*) embryos and tadpoles treated with triclopyr (2.33–19 µM at 48 hpf). However, the authors verified increased mortality after hatching due to organism sensitivity to triclopyr.

Triclopyr also induced yolk sac malabsorption at all concentrations tested in zebrafish embryos for up to 96 h. The yolk sac serves as a vital primary nutrient source for developing fish embryos: it supplies crucial energy reserves in the form of lipids and proteins for zebrafish to grow and to develop during the first five days post-fertilization (dpf), when the yolk sac is expected to be completely absorbed [38]. Yolk sac malabsorption during exposure to triclopyr may be linked to the interference of this herbicide in the nutrient transport from the yolk to the developing zebrafish embryo. This transport is mediated by lipoproteins within the yolk syncytial layer, which surrounds the yolk sac in zebrafish embryos [38]. If triclopyr negatively affects the function of the yolk syncytial layer, lipoproteins, or apolipoproteins, it may prevent the larvae from absorbing essential lipids from the yolk, to result in nutritional deficiencies and malabsorption [39]. Furthermore, alterations during early development, metabolic disorders, and behavioral changes in zebrafish may be related to the malabsorption of nutrients from the yolk sac [39], as observed in exposure to other auxin-like herbicides [35,36]. Therefore, we suggest that exposure to triclopyr may hinder normal availability and mobilization of the yolk sac content.

Defects in swim bladder development indicate an environmental risk related to exposure to contaminants [40]. In zebrafish, the swim bladder starts to inflate at 96 hpf [24]. In this study, we verified that the number of uninflated swim bladders at 96 hpf decreases irrespective of the tested triclopyr concentration. At 120 and 144 hpf, this decrease persists only at 50 µM triclopyr and is significant. Abnormal swim bladder development is a sublethal effect [41] that can influence aspects that are relevant to survival, such as feeding behavior, escape from predators, growth, and reproduction in fish [42].

Behavioral analysis is a reliable and highly responsive method to identify how chemicals impact fish. In the light/dark illumination exchange test, we observed a consistent pattern of increased motor activity during the dark phase. This pattern is described as anxious behavior. Nevertheless, when light is restored, zebrafish larvae return to their baseline swimming activity [43].

The acute exposure to sublethal concentrations of triclopyr decreased swimming activity in zebrafish larvae. During the behavioral test (light/dark transition), the larvae exhibited a baseline pattern during light periods and increased activity in the dark. However, triclopyr induced a decrease in the total distance at 10 µM during the first dark cycle, followed by a reduction in the total swimming distance at both 5 µM and 10 µM of triclopyr during the second dark period, when compared with the control group. Gaaied et al. [44] have documented a similar trend in zebrafish exposed to the lowest dose of 2,4-D (0.02 mg/L), an auxin-like herbicide closely associated with triclopyr [44]. Levin et al. [45] found that exposure to 100 ng/mL chlorpyrifos decreases swimming activity in zebrafish larvae at 120 hpf. Stehr et al. [30] reported reduced touch response in zebrafish embryos exposed to 10 mg/L (52 µM) clopyralid, an herbicide of the picolinic acid family, which also includes triclopyr. Furthermore, Berrill et al. [37] showed that the herbicide Garlon 4^®^, which contains triclopyr acid as an active ingredient, can alter behavior that impacts frog tadpole survival [37]. All these findings suggest that triclopyr may act as a neurotoxic compound, which supports the findings of the zebrafish motor activity assay conducted herein.

We performed the AChE activity assay to investigate whether triclopyr is neurotoxic. AChE plays a pivotal role in neural signal transmission—it breaks down the acetylcholine neurotransmitter. Additionally, AChE serves as a common biological marker for assessing exposure to neurotoxic compounds [46]. Compounds related to triclopyr, such as chlorpyrifos-methyl and its primary metabolite 3,5,6-trichloro-2-pyridinol, have been shown to inhibit AChE activity in zebrafish embryos [47]. Here, we verified that triclopyr does not inhibit or reduce AChE activity in zebrafish larvae after exposure for 96 h. In addition to AChE, other neurotransmitters such as dopamine, serotonin, acetylcholine, gamma-aminobutyric acid (GABA), glutamate, histamine, and glycine play crucial roles in motor activity [48]. Disruptions in the activity of these neurotransmitters can directly impact motor performance [49]; however, further investigations must be conducted to elucidate the mechanism of toxicity underlying the decrease in motor activity observed upon exposure to triclopyr.

In conclusion, studies on the toxicity of contaminants of emerging concern hold significant value and provide insights that help to predict potential short- and long-term impacts on environmental, animal, and human health. This study represents the first evidence that triclopyr adversely affects zebrafish early developmental stages. Fish, a component of only one trophic level in the aquatic environment, serve as valuable indicators when their early developmental stages are investigated in ecotoxicological tests. Such studies offer insights into potential consequences for species survival in future scenarios [50]. The effects of exposure to triclopyr observed in this study may also indicate how this herbicide impacts other trophic levels, highlighting risk to non-target aquatic organisms. Moreover, the data obtained here may be essential to support regulatory agencies in decision making regarding the assessment of potential impacts, development of risk management strategies, and establishment of continuous monitoring programs for this contaminant.

## 5. Conclusions

Our investigation has revealed that exposure to triclopyr results in abnormal embryonic development, characterized by reduced hatchability and yolk sac malabsorption. Moreover, although triclopyr did not inhibit AChE activity, swimming behavior was altered. The distinct behavioral response elicited by triclopyr underscores the complexity of its influence on zebrafish. These findings bear substantial implications for both ecological and human health, especially when we consider that triclopyr is present in the environment at concentrations evaluated in this study. Furthermore, this study has contributed to our understanding of how triclopyr influences zebrafish embryonic development and offered insights into its mechanisms of toxicity. Finally, given the pivotal role played by fish in the food chain, the consequences of exposure to triclopyr in terms of survival and population maintenance of the affected organisms are worrisome from an ecotoxicological standpoint.

## Data Availability

The data presented in this study are available on request from the corresponding author.
